# Heterotypic Tumor Spheroids in Agitation-Based Cultures: A Scaffold-Free Cell Model That Sustains Long-Term Survival of Endothelial Cells

**DOI:** 10.3389/fbioe.2021.649949

**Published:** 2021-06-09

**Authors:** Teresa Franchi-Mendes, Nuno Lopes, Catarina Brito

**Affiliations:** ^1^iBET, Instituto de Biologia Experimental e Tecnológica, Oeiras, Portugal; ^2^Instituto de Tecnologia Química e Biológica António Xavier, Universidade Nova de Lisboa, Oeiras, Portugal; ^3^The Discoveries Centre for Regenerative and Precision Medicine, Oeiras, Portugal

**Keywords:** 3D models, spheroids, endothelial cells, fibroblasts, breast cancer

## Abstract

Endothelial cells (ECs) are an important component of the tumor microenvironment, playing key roles in tumor development and progression that span from angiogenesis to immune regulation and drug resistance. Heterotypic tumor spheroids are one of the most widely used *in vitro* tumor microenvironment models, presenting improved recapitulation of tumor microenvironments compared to 2D cultures, in a simple and low-cost setup. Heterotypic tumor spheroid models incorporating endothelial cells have been proposed but present multiple limitations, such as the short culture duration typically obtained, the use of animal-derived matrices, and poor reproducibility; the diversity of culture conditions employed hinders comparison between studies and standardization of relevant culture parameters. Herein, we developed long-term cultures of triple heterotypic spheroids composed of the HCC1954 tumor cell line, human fibroblasts, and ECs. We explored culture parameters potentially relevant for EC maintenance, such as tumor cell line, seeding cell number, cell ratio, and agitation vs. static culture. In HCC1954-based spheroids, we observed maintenance of viable EC for up to 1 month of culture in agitation, with retention of the identity markers CD31 and von Willebrand factor. At the optimized tumor cell:fibroblast:EC ratio of 1:3:10, HCC1954-based spheroids had a higher EC area/total spheroid area at 1 month of culture than the other cell ratios tested. EC maintenance was tumor cell line-dependent, and in HCC1954-based spheroids it was also dependent on the presence of fibroblasts and agitation. Moreover, vascular endothelial growth factor (VEGF) supplementation was not required for maintenance of EC, as the factor was endogenously produced. ECs co-localized with fibroblasts, which accumulated preferentially in the core of the spheroids and secreted EC-relevant extracellular matrix proteins, such as collagen I and IV. This simple model setup does not rely on artificial or animal-derived scaffolds and can serve as a useful tool to explore the culture parameters influencing heterotypic spheroids, contributing to model standardization, as well as to explore molecular cross talk of ECs within the tumor microenvironment, and potentially its effects on drug response.

## Introduction

The tumor microenvironment (TME) impacts cancer progression, invasion, metastasis, and drug resistance (Quail and Joyce, 2013; Klemm and Joyce, 2015). The TME comprises several non-malignant cell types, of which the most represented are fibroblasts, immune and endothelial cells (ECs), and the non-cellular components, such as the extracellular matrix (ECM), soluble factors (Hanahan and Coussens, 2012; Quail and Joyce, 2013), and the characteristic physicochemical features, such as hypoxia and acidification (Schito and Semenza, 2016; Boedtkjer and Pedersen, 2020). In particular, ECs are major effectors of tumor angiogenesis, a recognized cancer hallmark, contributing to tumor growth, invasion, and metastasis (Bergers and Benjamin, 2003; Hanahan and Weinberg, 2011; Hanahan and Coussens, 2012). The progressive growth of the tumor eventually leads to intratumoral hypoxia, which induces hypoxia-inducible factor-1 (HIF-1) that in turn upregulates multiple target genes, such as the potent proangiogenic factor vascular endothelial growth factor (VEGF), promoting EC proliferation (Carmeliet, 2005; Schito and Semenza, 2016). Therapeutic agents targeting tumor angiogenesis, such as anti-VEGF or tyrosine kinase inhibitors, have been developed, initially with promising results; however, these therapies showed limited clinical success due to drug resistance and high toxicity, not predicted by the preclinical models employed (Bergers and Hanahan, 2008; Jain, 2014; Aalders et al., 2017). More recently, multiple studies suggested that ECs are modulated by the TME, acquiring genetic abnormalities and distinct transcriptional programs. These changes contribute not only to anti-angiogenic therapy resistance but also to functions behind the classical angiogenic role that are not observed in normal ECs. Tumor-associated ECs are now recognized as key mediators of immune regulation within the TME (Nagl et al., 2020), tumor metastization, and drug resistance mechanisms (Hida et al., 2018; Maishi et al., 2019). Endothelial to mesenchymal transition as a source of cancer-associated fibroblasts has also been reported (Liu et al., 2019). Besides tumor cells, non-malignant cells within the TME are also a source of proangiogenic cues (De Palma et al., 2017; Ireland and Mielgo, 2018); tumor-associated fibroblasts have been described to influence ECs by different mechanisms, including paracrine signaling, secretion of ECM proteins, and mechanotransduction (Kunz-Schughart et al., 2006; Hielscher et al., 2012; Sewell-Loftin et al., 2017). Nonetheless, modulation of ECs within the TME and their reciprocal cross talk with other cellular compartments are only partially unveiled (Hida et al., 2018; Ireland and Mielgo, 2018). On the other hand, the interest in ECs as a potential source of therapeutic targets has been revitalized. Therefore, well-defined and characterized cell models, in which EC interactions within the TME in different stages of tumor progression can be recapitulated, constitute a preclinical need.

Sophisticated organ-on-a-chip concepts are being explored for TME recapitulation (Michna et al., 2018; Park et al., 2019); however, these are not easily translatable into research labs and drug discovery settings. Heterotypic tumor spheroids are one of the most explored 3D models to incorporate ECs, as spheroids are easy to generate in large numbers and by a wide variety of methods, being cost-effective (Santo et al., 2017; Han et al., 2021). Typically, these spheroids include only tumor cells and ECs (Roudsari and West, 2016; Rodrigues et al., 2020). Additionally, most models described are short-term (Chen et al., 2009; Ingthorsson et al., 2010; Upreti et al., 2011; Correa de Sampaio et al., 2012; Aref et al., 2013; Asghar et al., 2015; Lamichhane et al., 2016; Chiew et al., 2017; Shoval et al., 2017; Lee et al., 2018) and require the use of animal-derived materials, such as basement membrane extracts from mouse sarcoma (BMEs, e.g., Matrigel^TM^, Benton et al., 2011, 2014). These BMEs are highly complex and poorly defined, rich in ECM proteins, growth factors, and cytokines, being a source of confounding factors and presenting batch to batch variability (Asghar et al., 2015). Moreover, the majority of the heterotypic spheroid models described were set up using distinct culture parameters, which hinders the comparison of model attributes and the choice of the adequate model system.

Herein, we aimed to develop a 3D TME spheroid model, simple to establish and to reproduce, in which the molecular cross talk between ECs and tumor cells, as well as other TME components, could be recapitulated. We hypothesized that the incorporation of fibroblasts in tumor cell—EC cocultures would improve TME recapitulation, resulting in the secretion of soluble factors and ECM proteins which are important for EC survival and function *in vivo*, potentially improving EC longevity in culture while avoiding the use of exogenous matrices. We established triple heterotypic spheroids in ultra-low attachment plates, composed of breast tumor cell lines, fibroblasts, and ECs, which were cultured for 30 days; we employed an orbital agitation-based culture system to facilitate mass transfer and improve the diffusion of those soluble factors (Santo et al., 2016). We evaluated key culture parameters that may influence long-term maintenance of ECs, namely, seeding cell number, cell ratio, and agitation vs. static long-term culture.

## Materials and Methods

### Cell Sources and 2D Cell Culture

The breast cancer cell lines MCF-7, BT474, HCC1954, HCC1806, and MDA-MB-231 were obtained from ATCC. MCF-7 and MDA-MB-231 cells were cultured in Dulbecco’s Modified Eagle Medium (DMEM) supplemented with 10% (v/v) fetal bovine serum (FBS) and 100 U/ml penicillin–streptomycin; for MCF-7, the medium was also supplemented with 4 mM Glutamax and 1 mM sodium pyruvate (all from Thermo Fisher, Waltham, MA, United States). BT474, HCC1954, and HCC1806 cells were cultured in Roswell Park Memorial Institute (RPMI) 1640 without phenol red, supplemented with 10% (v/v) FBS and 100 U/ml penicillin—streptomycin. For HCC1954 and HCC1806, the medium was further supplemented with 6 mM HEPES and 0.05 mM mercaptoethanol. Human dermal fibroblasts (hFs), from Innoprot, were cultured in Iscove’s Modified Dulbecco’s Medium (IMDM) supplemented with 10% (v/v) FBS and 1% (v/v) penicillin—streptomycin. Human umbilical vein endothelial cells (HUVECs, from now on referred to as ECs), acquired from Lonza (Basel, Switzerland), were maintained in Endothelial Cell Growth Medium 2 from PromoCell (Heidelberg, Germany) with 5 ng/ml epidermal growth factor, 10 ng/ml fibroblast growth factor 2 (FGF-2), 20 ng/ml insulin-like growth factor, 0.5 ng/ml VEGF, 1 μg/ml ascorbic acid, 22.5 μg/ml heparin, and 0.2 μg/ml hydrocortisone.

All cell types were expanded in adherent conditions, in an incubator at 37°C with a humidified atmosphere containing 5% CO_2_ in air. All tumor cells were subcultured twice a week, as previously described (Santo et al., 2016); hFs were passaged weekly, for up to 10–12 passages (Estrada et al., 2016). ECs were maintained according to the manufacturer’s instruction, subcultured once they reached 70–85% confluency, at a seeding density of 2,500 cell/cm^2^.

### 3D Cell Culture

Each tumor cell line was mixed with hF and ECs as single-cell suspensions and seeded in ultra-low adherence (ULA) 96-well plates (Corning, Corning, NY, United States) to generate heterotypic cell spheroids. After 2 days of aggregation, spheroids were transferred to shake flasks under continuous orbital shaking at 100 rpm and cultured for an additional 28 days (Supplementary Figure 1). All cultures were maintained in Endothelial Cell Growth Medium 2 from PromoCell with 10% (v/v) FBS, with a 50% medium exchange every 3–4 days. For the screening of the different breast cancer cell lines, 6,000 cells were plated per well, at a tumor cell:hF:EC ratio (from here on referred to as TC:hF:EC) of 1:1:1. For HCC1954 studies, multiple conditions were tested: (i) seeding total cell numbers of 1,000, 3,000, and 6,000 cell/well and (ii) TC:hF:EC ratios of 1:1:1, 1:1:3, 1:1:10, 1:3:3, 1:3:5, and 1:3:10; long-term culture in static vs. agitation conditions; and VEGF supplementation (0.5 ng/ml). In some of the experiments, ECs were labeled with the fluorescent dye CellTracker^TM^ Deep Red (Invitrogen, Carlsbad, CA, United States) and/or hFs were labeled with the PKH26 Red Fluorescent Cell Linker Kit (Sigma-Aldrich, St. Louis, MO, United States) before seeding for spheroid aggregation; labeling was performed according to the manufacturer’s instructions and as described before (Rebelo et al., 2018). Spheroids were characterized at days 2, 9, 14, 16, and 30 days of culture.

### Cell Viability

Cell viability was assessed through a fluorescent membrane integrity assay to discriminate between live and dead cells. Spheroids were incubated with 10 μg/ml of fluorescein diacetate (FDA; Sigma-Aldrich) and 2 μg/ml of propidium iodide (PI; Sigma-Aldrich) and were observed under a fluorescence microscope (DMI6000B, Leica Microsystems, Wetzlar, Germany). Cells that accumulated the fluorescent metabolization product of FDA were considered alive, while cells positive for PI staining were considered dead.

### Spheroid, EC, and hF Area Quantification

The spheroid area was defined as the FDA-positive area in the equatorial plane of the spheroid; EC and hF area as the Cell Tracker^TM^ Deep Red- and PHK26-positive areas, respectively. The positive regions were identified by applying automated threshold adjustment in FIJI open-source software, and the areas were determined by the measurement algorithm (Rasband, WS, ImageJ, US National Institutes of Health, Bethesda, MD, United States^1^, 1997–2012). EC and hF areas were normalized by the total spheroid area.

### Immunofluorescence Microscopy and Image Analysis

Samples were collected from culture at day 30 and fixed in 4% (v/v) paraformaldehyde (PFA)/4% (v/v) sucrose for 20 min. Spheroids were dehydrated with 30% (w/v) sucrose overnight, frozen at –80°C in Tissue-Tek O.C.T. (Sakura, Alphen aan den Rijn, Netherlands) and sectioned at 10 μm thickness using a cryomicrotome (Cryostat I, Leica). Immunofluorescence was performed according to previously published methods (Rebelo et al., 2015; Simão et al., 2015). Briefly, cells were permeabilized with 0.1% Triton X-100 (w/v) and blocked in 1% bovine serum albumin (BSA). Primary antibodies were diluted in 1% (w/v) BSA and incubated overnight at 4°C. Secondary antibodies were diluted in 1% (w/v) BSA and incubated for 1 h at RT. Samples were mounted in ProLong Gold Antifade Mountant containing DAPI (Thermo Fisher) and visualized using a fluorescence microscope (DMI6000B, Leica Microsystems). The primary antibodies used were anti-CD31, von Willebrand factor (vWF), collagen types I and IV, and fibronectin (all from Abcam, Cambridge, United Kingdom).

### Quantification of Soluble Factors

Culture supernatants were collected and centrifuged at 1,000 × g for 5 min. Samples were snap-frozen and stored at –80°C until analysis. The Human VEGF Quantikine and Interleukin-6 (IL-6) Quantikine ELISA Kits (all from R&D Systems, Minneapolis, MN, United States) were employed according to the manufacturer’s instructions. Absorbance was measured in microplate reader Infinite^®^ 200 PRO (NanoQuant, Tecan Trading AG, Männedorf, Switzerland).

### Statistical Analysis

Statistical analysis was carried out using GraphPad Prism 8 software. Data were collected from at least 10–50 spheroids from three independent experiments as mean ± SD, unless noted otherwise. Statistical tests are detailed in each figure legend, and all significant differences are indicated in the graphs.

## Results

### Maintenance of EC in Triple Heterotypic Spheroids Is Tumor Cell Line Dependent

**FIGURE 1:**
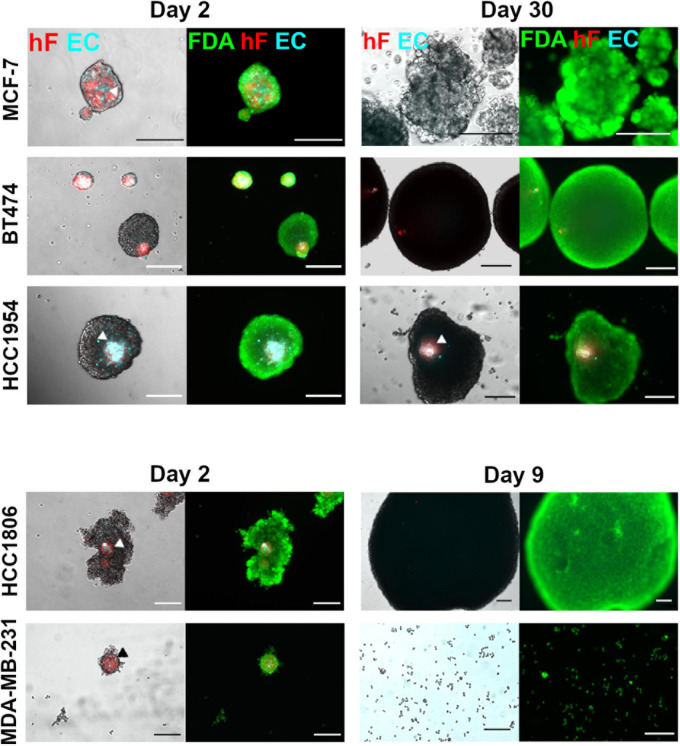
Maintenance of ECs in triple heterotypic spheroids is tumor cell line dependent. Phase contrast and fluorescence microscopy of triple heterotypic spheroids in agitation-based cultures, at day 2 (after transfer to shake flasks) and day 30 of culture (after 2 days of aggregation in static culture followed by 28 or 7 days of agitation-based culture). HCC1806 and MDA-MB-231 spheroid cultures were terminated at day 9 (day 7 in agitation-based culture). Spheroids of breast cancer cell lines (TC); human dermal fibroblasts, labeled with PKH26 (hF, red); HUVEC, labeled with CellTracker Deep Red (EC, cyan), in a TC:hF:EC ratio of 1:1:1 (6,000 cells per well). Live cells were labeled with FDA (green). White and black arrowheads identify EC and hF in triple heterotypic spheroids. Images are representative of the 5–10 spheroids assessed per condition, in each of the three independent experiments performed (*N* = 3). Scale bar, 250 μm.

Five breast cancer cell lines were employed to generate heterotypic spheroids of tumor cells, fibroblasts, and ECs, in ULA 96-well plates. After 2 days of aggregation, distinct spheroid formation efficiency and spheroid morphology were observed for each of the cell lines (Figure 1 and Supplementary Figures 2A–C). The BT474 cell line formed compact spheroids from which fibroblasts and ECs were precluded (Figure 1 and Supplementary Figures 2A,B); stromal cells formed independent spheroids, resulting in two distinct spheroid populations with different sizes (Supplementary Figure 2B). For the remaining cell lines tested, one heterotypic spheroid *per* well was obtained, composed of the three cell types, in which fibroblasts and ECs co-localized preferentially in the spheroid center (Figure 1 and Supplementary Figure 2A, arrowheads). This spatial distribution was independent of spheroid compaction, measured by visual inspection of cell packing density (Santo et al., 2016). HCC1954 cells formed compact spheroids, MCF-7 cells formed spheroids that presented loosely attached cells at their surface, and HCC1806 and MDA-MB-231 cells formed cell clumps of irregular shape, with a more compact center (Figure 1 and Supplementary Figure 2A).

After a 2-day aggregation period, spheroids were transferred into shake flasks and thereafter cultured under orbital agitation, to improve gas and mass transfer within the spheroid (Serra et al., 2012; Schmelzer et al., 2015; Santo et al., 2016). By day 30 of culture, only the HCC1954 spheroids retained the three cell components, with EC and fibroblasts detected in the spheroid core (Figure 1). Furthermore, these spheroids exhibited a significant increase in total area from days 2 to 30 (2.0-fold increase, Supplementary Figure 2D). For BT474, spheroids retained the circular morphology (Figure 1), with a significant area increase of 13.6-fold from days 2 to 30 (Supplementary Figure 2D); these results suggest tumor cell proliferation. Nonetheless, fibroblasts and ECs were not detected in culture by day 30 (Figure 1), as confirmed by the absence of CD31-positive cells, an endothelial marker (Miettinen et al., 1994; Supplementary Figure 2E). For MCF-7 spheroids, fibroblasts and ECs were not detected as well at day 30 (Figure 1); regarding the total spheroid area, MCF-7 spheroids showed a modest 1.4-fold increase from days 2 to 30 of culture (Supplementary Figure 2D). Regarding HCC1806 and MDA-MB-231-based cultures, spheroids could not be maintained for more than a few days; from days 2 to 9 of culture, the HCC1806 spheroid area increased (Figure 1) and the spheroid number decreased steeply (data not shown), suggesting spheroid fusion; MDA-MB-231 spheroids dissociated progressively and, by day 9, the culture was mainly composed by individual cells or in small clumps (Figure 1). For these two cell lines, EC and fibroblasts were not detected by day 9. Therefore, these cultures were terminated on day 9 and not further pursued. In summary, HCC1954 heterotypic spheroids generated in ULA plates exhibited EC-positive areas by day 30 of culture, whereas for the other breast cancer cell lines tested the EC population did not persist. Therefore, the HCC1954 cell line was selected to address the influence of distinct cell culture parameters on the long-term maintenance of ECs in heterotypic spheroids.

### ECs and Fibroblasts Co-localize in HCC1954-Based Triple Heterotypic Spheroids

**FIGURE 2:**
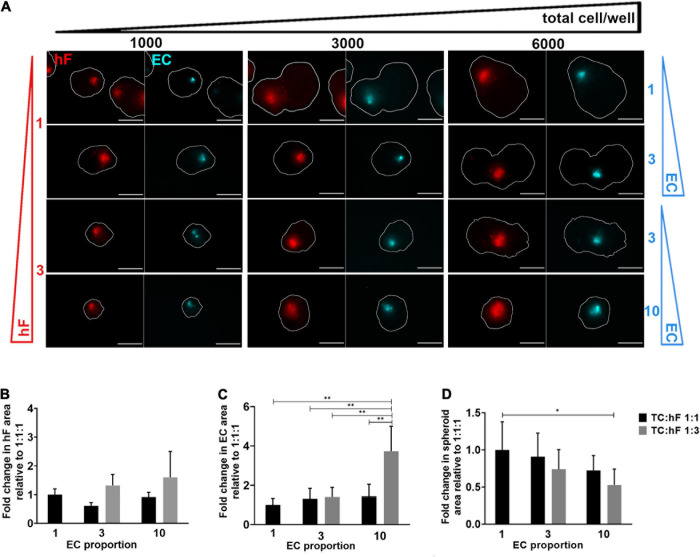
Fibroblast and endothelial cell content in triple heterotypic spheroids: effect of total cell number and cell ratio. **(A)** Fluorescence microscopy of spheroids at day 30 of culture (after 2 days of static culture followed by 28 days of agitation-based culture). Spheroids of HCC1954 breast tumor cells (TC); human dermal fibroblasts, labeled with PKH26 (hF, red); HUVEC, labeled with CellTracker Deep Red (EC, cyan), seeded at the TC:hF:EC ratios and cells per well indicated. The spheroid outline at the equatorial axis is represented in a solid white line and was determined based on the FDA labeling of live cells (Supplementary Figure 3A). Images are representative of the 5–10 spheroids assessed per condition, in each of the three to four independent experiments performed. Scale bar, 250 μm. Quantification of fold change in **(B)** hF area, **(C)** EC area, and **(D)** total spheroid area at the equatorial axis, for spheroids from A, seeded at 6,000 cells per well. The EC and hF areas were normalized for the total spheroid area at the equatorial axis; the fold change was determined relative to the condition with a TC:hF:EC of 1:1:1. Data are presented as mean ± SD from *N* = 3–4 (independent experiments); in each experiment, at least 20 spheroids were quantified per condition; statistical analysis was performed using a one-way ANOVA followed by a *post hoc* Tukey’s multiple-comparison test; **p < 0.01, *p < 005.

HCC1954 triple heterotypic spheroid formation was further explored to assess the influence of distinct culture parameters on long-term EC maintenance. In a first step, seeding cell number (total cells *per* well) and TC:hF:EC ratio were evaluated. The main readouts were cell viability and EC area (the area identified as the CellTracker^TM^ Deep Red-positive, normalized by the total spheroid area, defined as FDA-positive). For all ratios tested, approximately 20% of the spheroids were composed only of tumor cells (data not shown). For a seeding of 6,000 cell/well, there was a tendency for higher EC area at day 30 of culture, which was up to 2.7—higher than for the 1,000 cell/well condition (Figure 2A and Supplementary Figures 3A,B)—despite some degree of intrinsic variability observed, especially for the lower seeding densities. Therefore, these lower seedings (1,000 and 3,000 cell/well) were not further explored.

For 6,000 cell/well, the TC:hF:EC ratio of 1:3:10 led to a significantly higher EC area than the 1:1:1 ratio (approximately 3.7-fold, ^∗∗^*p* < 0.01, Figure 2C) and all other ratios tested (*p* < 0.01, Figure 2C); no significant differences in EC area were found between the other cell ratios. The hF area was also measured, and no significant differences were found between conditions (Figure 2B). Preliminary data from flow cytometry detection for EC (CellTracker^TM^ Deep Red-positive cells) also suggested a trend toward a higher EC fraction in spheroids seeded at 1:3:10 than at a 1:1:1 ratio, in both timepoints (Supplementary Figure 4). Of notice, at day 2 the total spheroid area was similar for the different cell ratios tested (Supplementary Figure 5A) but at day 30, the spheroids seeded at 1:1:1 presented up to a 1.9-fold increase in area, when compared to other cell ratios (Figure 2D and Supplementary Figure 5A). The conditions with a higher proportion of tumor cells led to the generation of larger spheroids (Figure 2D), probably due to the proliferative behavior typical of tumor cells (Hanahan and Weinberg, 2011). The presence of stromal cells seemed to attenuate this effect, as observed by the evident increase along time in the total spheroid area of tumor homotypic cultures, compared to the heterotypic 1:3:10 spheroids generated with the same number of tumor cells (Supplementary Figures 5B,C). Heterotypic spheroids seeded at 1:1:1 and 1:3:10, and homotypic tumor spheroids showed high cell viability, as observed by detection of live and dead cells, assessed by FDA and PI, respectively (Supplementary Figure 5C).

**FIGURE 3:**
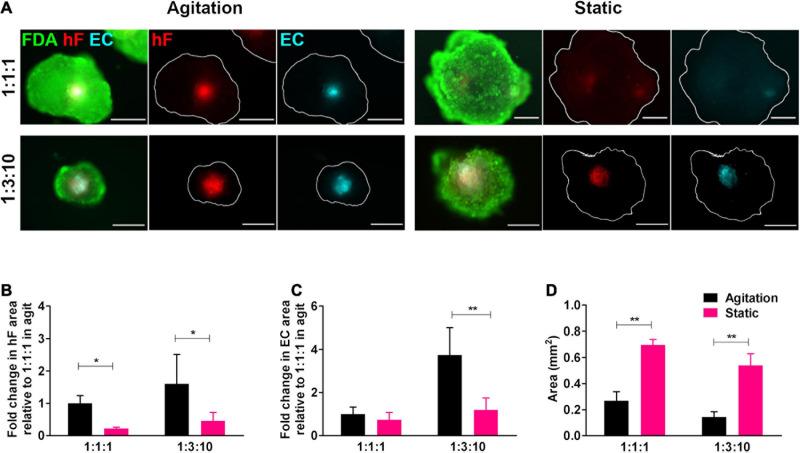
Cell viability and endothelial cell content in triple heterotypic spheroids: effect of agitation. **(A)** Fluorescence microscopy of triple heterotypic spheroids at day 30 of culture in agitation (2 days of static culture followed by 28 days of agitation-based culture, rotational speed of 100 rpm) or in static (30 days of static culture) conditions. Spheroids generated by seeding 6,000 cells per well, at distinct ratios of HCC1954 breast tumor cells (TC); human dermal fibroblasts, labeled with PKH26 (hF, red); and HUVEC, labeled with CellTracker Deep Red (EC, cyan). Live cells were stained with FDA (green). Images are representative of the 5–10 spheroids assessed per condition, in each of the three to four independent experiments performed. Scale bar, 250 μm. Quantification of **(B)** fold change in hF area, **(C)** fold change in EC area, and **(D)** total spheroid area at the equatorial axis, for spheroids from A. The EC and hF areas were normalized for the total spheroid area at the equatorial axis; the fold change was determined relative to the condition with a TC:hF:EC of 1:1:1, agitation. Data are presented as mean ± SD from *N* = 3–4 (independent experiments); in each experiment, at least 20 spheroids were quantified per condition. Statistical analysis was performed using unpaired *t*-test two-tailed comparing agitation to static for each cell ratio; in B, *p = 0.015 for 1:1:1 and *p = 0.047 for 1:3:10; in C, **p = 0.0068 for 1:3:10; and in D, **p = 0.0017 for 1:1:1, **p = 0.0014 for 1:3:10.

To ascertain the effect of agitation on the long-term viability of ECs in triple heterotypic spheroids, dynamic culture was compared with a static culture control. On day 30 of culture, fibroblast-positive areas were detected by the PKH26 signal and EC-positive areas by the CellTracker^TM^ Deep Red signal and by immunodetection of the endothelial marker CD31 (Figure 3A and Supplementary Figure 6). For both static and dynamic conditions, fibroblasts and ECs organized preferentially in the spheroid center (Figure 3A). The hF relative area was significantly higher in dynamic conditions than in static, at both cell ratios tested (^∗^*p* < 0.05, Figure 3B). For spheroids generated at 1:3:10, EC-positive areas were detected in both agitation and static conditions (Figure 3A and Supplementary Figure 6) but the EC relative area was significantly higher in dynamic cultures (3.1-fold higher than static, ^∗∗^*p* = 0.0068, Figure 3C). This was mainly due to the higher total spheroid area observed in static conditions, with an approximately 3.8-fold increase relative to orbital agitation (Figure 3D). For spheroids generated at 1:1:1 in static conditions, EC-positive areas were small or absent (Figure 3A and Supplementary Figure 6). Overall, the results pointed to the beneficial effect of agitation in sustaining ECs in heterotypic spheroids.

**FIGURE 4:**
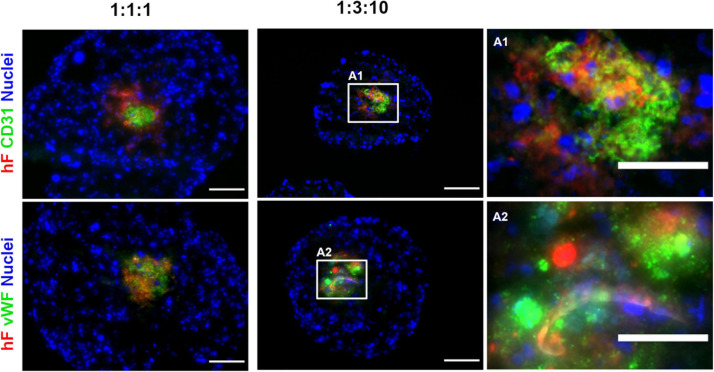
Endothelial cells maintained their identity and phenotype in triple heterotypic spheroids after 30 days of culture. Immunofluorescence microscopy on cryosections of spheroids, collected at day 30 (2 days of static culture followed by 28 days of agitation-based culture, rotational speed of 100 rpm). Detection of the EC markers, CD31 (green) and von Willebrand factor (vWF, green); nuclei labeled with DAPI (blue). Spheroids generated by seeding 6,000 cells per well of HCC1954 breast tumor cells (TC), human dermal fibroblast, labeled with PKH26 (hF, red); HUVEC (EC), at TC:hF:EC ratios of 1:1:1 and 1:3:10. Images are representative of the 5–10 spheroids assessed per condition, in each of the three independent experiments performed. Left and middle panels: scale bar, 100 μm; right panel **(A1,A2)** are high-magnification insets of the regions indicated by the white squares in the middle panel: scale bar, 50 μm.

To confirm the phenotype of EC in long-term cultures, specific lineage markers, namely, CD31 and vWF, were evaluated by immunofluorescence, on day 30. CD31- and vWF-positive cells were always detected, indicating that ECs retained their identity along 1 month of culture. ECs were in close vicinity to fibroblasts, in the center of the spheroids (green and red, respectively, Figures 4A1,2). In fact, regardless of the cell ratio and the seeding cell number tested, ECs and fibroblasts were localized in close vicinity within the core of the triple heterotypic spheroid (Figures 1A, 2A, 3A, 4), suggesting a possible role for fibroblast on EC support.

### Fibroblasts Are Required to Maintain the EC Population

**FIGURE 5:**
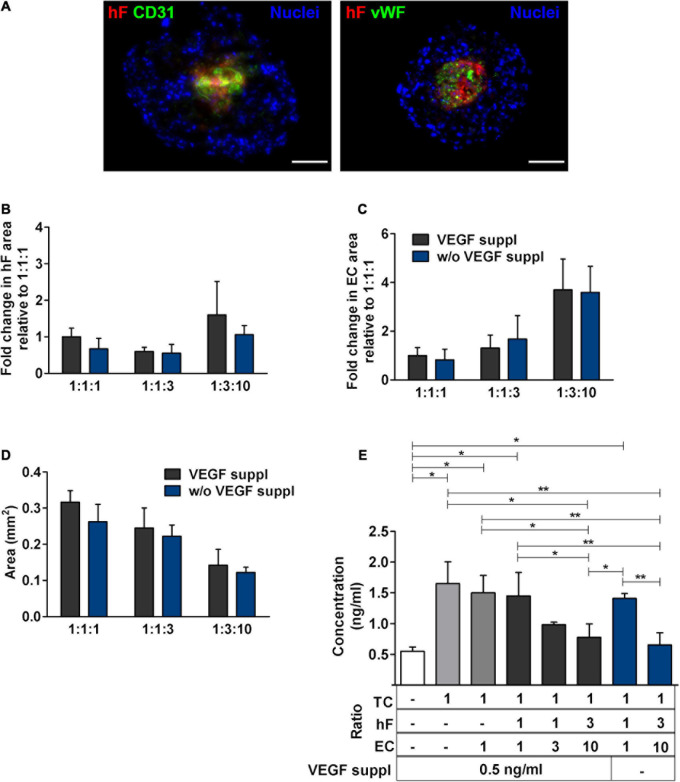
Medium supplementation with vascular endothelial growth factor (VEGF) was not required for EC maintenance in triple heterotypic spheroids. **(A)** Immunofluorescence microscopy on cryosections of spheroids, collected at day 30 of culture (2 days of static culture followed by 28 days of agitation-based culture, rotational speed of 100 rpm). Detection of the EC markers, CD31 (green) and von Willebrand factor (vWF, green), in spheroids cultured with supplementation of VEGF (0.5 ng/ml), or without (w/o VEGF supplementation); nuclei were labeled with DAPI (blue). Spheroids generated by seeding 6,000 cells per well of HCC1954 breast tumor cells (TC); human dermal fibroblasts, labeled with PKH26 (hF, red); HUVEC (EC), at a TC:hF:EC ratio of 1:3:10. Images are representative of the 5–10 spheroids assessed per condition, in each of the three independent experiments performed. Scale bar, 100 μm. Quantification of **(B)** fold change in hF area, **(C)** fold change in EC area, and **(D)** total spheroid area at the equatorial axis, for spheroids from A. The EC and hF areas were normalized for the total spheroid area at the equatorial axis; the fold change was determined relative to the condition with a TC:hF:EC ratio of 1:1:1, with VEGF. Data are presented as mean ± SD from *N* = 3–4 (independent experiments); in each experiment, at least 20 spheroids were quantified per condition. Statistical analysis was performed using unpaired *t*-test two-tailed comparing VEGF supplemented to VEGF not supplemented for each cell ratio; no statistical significance was found. **(E)** VEGF concentration in the spheroid culture supernatants from day 30, quantified by ELISA. White bar represents fresh culture medium. Data presented as mean ± SD from three to four independent experiments (*N* = 3–4). Statistical analysis was performed by one-way ANOVA followed by a *post hoc* Tukey’s multiple-comparison test; *p < 0.05; **p < 0.01; for each ratio, no statistical significance was found between conditions with and without VEGF supplementation at each cell ratio.

The proximity between fibroblasts and ECs led us to hypothesize that fibroblasts played a role in EC preservation in the triple heterotypic spheroids. In fact, in double spheroids composed of HCC1954 cells and EC, with TC:EC ratios of 1:1 and 1:10, the EC signal was nearly non-detected by day 30 (Supplementary Figure 7A), which was corroborated by the absence of CD31-positive cells (data not shown). Tumor-associated fibroblasts are known to secrete proangiogenic growth factors such as VEGF, one of the most prominent and potent (De Palma et al., 2017), which is regularly used as a supplement for EC culture and was a component of the medium used for triple heterotypic spheroids. Therefore, we asked if indeed fibroblasts were secreting additional VEGF, required for EC survival. Triple spheroids generated at different cell ratios were cultured in the medium without VEGF supplementation for 30 days, after which the EC phenotype, total spheroid area, EC relative area, and VEGF concentration in the conditioned medium were assessed. Monotypic tumor spheroids and tumor–EC double spheroids were cultured as controls. In spheroids cultured without VEGF supplementation, the CD31- and vWF-positive cells were detected mainly in the center of the spheroids, co-localizing with the fibroblasts, in a pattern similar to what was observed with VEGF supplementation (Figures 5A, 4, respectively); hF and EC relative areas and total spheroid area were also similar, with no significant differences between the conditions with or without VEGF, for each cell ratio (Figures 5B–D). The VEGF concentration was also similar in supplemented and non-supplemented cultures of each cell ratio (e.g., 0.78 ± 0.19 and 0.65 ± 0.17 ng/ml, for the 1:3:10 ratio, with and without supplementation, respectively, Figure 5E), and always higher than the 0.5 ng/ml of the fresh supplemented medium. On the other hand, the VEGF concentration was significantly higher in cultures of spheroids with a larger proportion of tumor cells (1:1:1 vs. 1:3:10, with or without VEGF supplementation, Figure 5E), even in the absence of fibroblasts (monotypic tumor spheroids and tumor–EC double heterotypic spheroids, Figure 5E); nonetheless, ECs were not maintained in these double cocultures (Supplementary Figure 7A). Thus, these results indicated that VEGF supplementation (0.5 ng/ml) did not impact EC maintenance in heterotypic spheroids and that even higher concentrations of this factor (up to 1.5 ng/ml) were not sufficient to sustain ECs in the absence of fibroblasts. Moreover, the data suggested that VEGF was mainly produced by HCC1954 tumor cells and that it was consumed by ECs in spheroids with higher proportions of these cells (1:1:3 and 1:3:10). We have also assessed IL-6, a proinflammatory cytokine secreted by tumor cells and fibroblasts, involved in angiogenesis (Nagasaki et al., 2014; Hegde et al., 2020). IL-6 appeared to be secreted by tumor cells and by fibroblasts and consumed by ECs (Supplementary Figure 8); although further studies will be required to conclude on the role of IL-6, its concentration was not correlated with EC maintenance. Furthermore, the conditioned medium from fibroblasts was not sufficient to sustain ECs in double spheroids with tumor cells (Supplementary Figure 7B), which suggests that other fibroblast-derived factors were contributing to EC maintenance.

**FIGURE 6:**
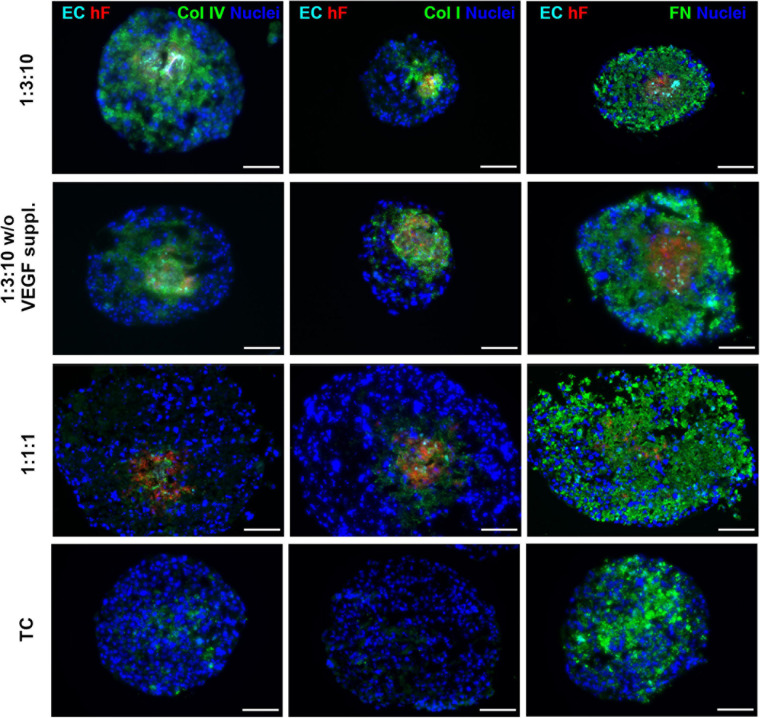
Triple heterotypic spheroids were enriched in ECM proteins. Immunofluorescent detection of collagen IV (Col IV), collagen I (Col I), and fibronectin (FN), all in green; nuclei were labeled with DAPI (blue). Spheroids generated by seeding 6,000 cells per well of HCC1954 breast tumor cells (TC), human dermal fibroblasts, labeled with PKH26 (hF, red); HUVEC, labeled with CellTracker Deep Red (EC, cyan), at a TC:hF:EC ratio of 1:1:1 and 1:3:10. Spheroids were cultured with the standard supplementation of VEGF (0.5 ng/ml), or without (w/o VEGF supplementation) and analyzed at day 30 of culture (2 days of static culture followed by 28 days of agitation-based culture, rotational speed of 100 rpm). Images are representative of the 5–10 spheroids assessed per condition, in each of the three independent experiments performed (*N* = 3). Scale bar, 100 μm.

Specific ECM proteins, such as collagen I and IV and fibronectin, have been described as supportive for ECs within the TME (Lu et al., 2012; Fang et al., 2014; Pickup et al., 2014) and are known to be secreted by fibroblasts (Kalluri and Zeisberg, 2006; Newman et al., 2011). Therefore, we investigated the presence of these ECM proteins in heterotypic spheroids after 30 days of culture. Fibronectin was detected both in triple and in monotypic spheroids, widely distributed throughout the entire spheroid area (Figure 6), suggesting that this protein was mainly secreted by tumor cells. Collagen I and IV were strongly detected in triple heterotypic spheroids, whereas in homotypic tumor spheroids collagen IV was faintly detected and collagen I was not detected (Figure 6). Detection of both collagens was higher in spheroids generated with a 1:3:10 ratio (with or without VEGF supplementation) than in 1:1:1 (Figure 6), and particularly enriched in the center of the spheroid where ECs and fibroblasts were present (Figure 6). The results suggest that collagen I and IV may be important fibroblast-derived factors providing support to ECs.

## Discussion

ECs play a major role in tumor angiogenesis, being critical in fueling tumor growth and progression (Hanahan and Coussens, 2012). EC-targeting therapy, such as anti-angiogenic strategies, have shown promising results yet have failed to reach in the clinics the success observed in animal models (Ye, 2016). Predictive biomarkers are still required to stratify which patients will benefit from this therapy (Aalders et al., 2017). Additionally, other EC-targeting approaches are under development, such as disruption of EC metabolism (Cantelmo et al., 2016). Moreover, ECs actively contribute to immune regulation, metastasis, and drug resistance, by interaction with tumor cells but also with other non-malignant cells of the TME, particularly immune cells and fibroblasts (Stacer et al., 2016; Cao et al., 2017; Nagl et al., 2020). Overall, EC inclusion in 3D *in vitro* models constitutes a step closer toward understanding heterotypic cross talk influencing processes from tumor progression to metastatic setting.

Here, we developed triple heterotypic spheroids of breast cancer cells, fibroblasts, and ECs, all from human sources, without resourcing to artificial or animal-derived scaffolds, taking advantage of the intrinsic tumor cell ability to aggregate; the ability of the different cell components to secrete ECM and soluble factors that are proangiogenic; and the improved mass transfer achieved in agitation-based culture systems. We explored this simple setup to study different culture parameters, assessing their effect on the long-term maintenance of ECs. Multiple *in vitro* studies have been performed, aiming to represent ECs within the TME, many of them employing the spheroid approach (Walter-Yohrling et al., 2003; Upreti et al., 2011; Correa de Sampaio et al., 2012; Ehsan et al., 2014; Lamichhane et al., 2016; Amann et al., 2017; Chiew et al., 2017; Shoval et al., 2017; Lee et al., 2018). However, most of these studies rely on the use of animal-derived materials or murine cells (Walter-Yohrling et al., 2003; Ingthorsson et al., 2010; Upreti et al., 2011; Seano et al., 2013; Lee et al., 2018). Furthermore, the average culture time is short, especially when using microfluidic devices (Aref et al., 2013; Asghar et al., 2015; Michna et al., 2018), which can hinder the recapitulation of the long-term effects of the cell interactions within the TME on tumor progression or drug response (Loessner et al., 2010; Unger et al., 2014; Estrada et al., 2016). Another confounding factor when comparing data generated in these spheroid models or selecting an adequate cell model setup is the vast diversity in important culture parameters employed in different cell models.

We investigated different breast cancer cell lines and observed distinct abilities to form spheroids and distinct morphologies, which have previously been linked to intrinsic tumor cell line characteristics (Friedrich et al., 2007, 2009). Our data in ULA plates corroborate previous studies reporting heterotypic tumor-fibroblast spheroid formation in different platforms. BT474 tumor cells and fibroblasts formed independent spheroids (Kunz-Schughart et al., 2001); in the case of MCF-7 cells, fibroblasts also localized in the center of the spheroid with tumor cells distributed in the periphery (Angelucci et al., 2012; Stock et al., 2016). Ehsan et al. reported different migration behaviors depending on the tumor cell line used to set up triple cultures of tumor, fibroblasts, and ECs in a fibrin gel (Ehsan et al., 2014).

After aggregation, spheroids were transferred to orbital agitation; we observed maintenance of ECs for 1 month in culture only when using the HCC1954 cell line, suggesting that this phenomenon is cell line specific, as it was also observed by others that different tumor cell lines differ in spheroid morphology (Österholm et al., 2012), adhesion marker expression (Stadler et al., 2018), and EC-promoting capabilities (Chiew et al., 2017; Shoval et al., 2017). We confirmed EC identity by detection of specific markers by day 30 of culture. Positivity for CD31 and vWF ruled out endothelial to mesenchymal transition to fibroblast-like cells, which has been previously described (Zeisberg et al., 2007; Potenta et al., 2008).

EC detection by day 30 of culture was always associated with the presence of fibroblasts, suggesting that the latter might be sustaining ECs in HCC1954 heterotypic spheroids. Fibroblasts have been reported to support ECs whether in tumor (Janvier et al., 1997; Walter-Yohrling et al., 2003; Vong and Kalluri, 2011) or in other pathologies, as well as in the healthy tissue context (Newman et al., 2011; Costa-Almeida et al., 2015). *In vitro*, co-localization of ECs and fibroblasts has been reported, whether using 3D models of lung cancer (Amann et al., 2017) and pancreatic cancer (Lazzari et al., 2018). This trend was also observed in another study using mesenchymal stromal cells and human pulmonary microvascular endothelial cells for the formation of spheroids with a lung cancer cell line (Lamichhane et al., 2016). Other reports observed tube-like structures only in the presence of fibroblasts (from murine source), using a microfluidic device with the A549 lung tumor cell line, embedded in a collagen I gel (Amann et al., 2017; Lee et al., 2018). Furthermore, Hielscher et al. (2012) reported that ECMs secreted from MCF-7 or MDA-MB-231 cells were not sufficient to support EC migration into tube-like structures, only when combined with fibroblast-derived ECM. Curiously, even fibroblasts derived from the same source can exhibit subpopulations with distinct abilities to promote EC tube-like structures in double cocultures *in vitro* (Sorrell et al., 2008).

We hypothesize that fibroblast support to ECs was through secretion of essential soluble factors and/or structural proteins, as it is also described for other cell types of mesenchymal origin (Gerhardt and Betsholtz, 2003; Potapova et al., 2007). Regarding specific ECM components, we observed collagen I and IV in the triple heterotypic spheroids but not in tumor monocultures, which suggests that these proteins were derived from fibroblasts. However, we cannot exclude that ECs were also providing ECM, as ECs and fibroblasts co-localized in our triple spheroids and HUVECs are reported to produce fibronectin and collagen IV (Kusuma et al., 2012). Nonetheless, mono-spheroids of ECs dissociated once transferred to shake flasks (data not shown) and we have previously demonstrated secretion of collagen I by hFs in coculture with tumor cells (Estrada et al., 2016). Collagen I and fibronectin as scaffolds for EC tube-like formation *in vitro* are well documented (Davis et al., 2002; Sweeney et al., 2003; Whelan and Senger, 2003; Davis and Senger, 2005), although other fibroblast-derived proteins can be involved (Newman et al., 2011), and matrix composition is reported to influence EC growth and support (Kroon et al., 2002; Rao et al., 2012; Feng et al., 2013). The fact that the fibroblast-conditioned medium was not sufficient for EC maintenance, similar to what has been reported by others (Newman et al., 2011), also corroborates the hypothesis that deposited ECM proteins secreted by fibroblasts were playing an important role in EC survival. Interestingly, VEGF medium supplementation was not a requirement for EC long-term survival in the optimized cell ratio, which represents an advantage when setting up preclinical models, avoiding batch-to-batch variation of recombinant growth factors and reducing costs. VEGF has a prominent proangiogenic role, inducing EC proliferation and migration (Amann et al., 2017). Our data suggest that the VEGF produced within spheroids, by tumor cells, was taken up by ECs, recapitulating endogenous cellular cross talk and proangiogenic signaling within the TME. In parallel, we detected secretion of IL-6 probably by tumor cells and fibroblasts; this pro-inflammatory cytokine, secreted by cancer-associated fibroblasts and tumor cells, also has a pro-angiogenic role and has been reported to be increased in the plasma of breast cancer patients and linked to higher tumor stage (Salgado et al., 2003; Ravishankaran and Karunanithi, 2011). An immediate useful application of the model setup is the systematic identification of the ECM proteins and soluble factors involved in the cellular cross talk responsible for the maintenance of EC viability and phenotype.

Interestingly, in the agitation-based culture, we observed larger EC relative areas in comparison to static conditions. Our data highlights the relevance of using dynamic culture systems with improved gas and mass transfer that can better represent in-tissue soluble factor distribution than static cultures (Serra et al., 2012; Schmelzer et al., 2015; Santo et al., 2016). Agitation also brought the additional advantage of controlling spheroid size, with spheroids reaching significantly higher areas in static conditions than under agitation. As monocultures of tumor cells also showed a higher total area than triple spheroids, together these observations suggest that tumor cells in monocultures are proliferation-driven, while the presence of stromal cells and the factors they secrete can be redirecting tumor cells to a less proliferative and more invasion-oriented phenotype (Hinz et al., 2001; Grinnell, 2003; Doyle et al., 2015).

In summary, we propose a simple methodology to generate and maintain for long periods (at least up to a month) heterotypic spheroids. We can envision the inclusion of other cell types of the TME, namely, immune cells. This setup can be a useful tool to explore the culture parameters influencing heterotypic spheroids, contributing to model standardization, as well as to explore the molecular cross talk of ECs within the TME, and potentially its effects on response to long-term drug exposure, without the confounding factors of exogenous matrices.

## Data Availability Statement

The raw data supporting the conclusions of this article will be made available by the authors, without undue reservation.

## Author Contributions

TF-M and CB: study conceptualization, experimental design, data analysis, and data interpretation. TF-M and NL: data acquisition. TF-M: first manuscript draft. TF-M, NL, and CB: manuscript revision and editing. All the authors read and approved the final manuscript.

## Conflict of Interest

The authors declare that the research was conducted in the absence of any commercial or financial relationships that could be construed as a potential conflict of interest.

## Footnotes

1 http://imagej.nih.gov/ij/
