# Supplementary material for: Heterotypic Tumor Spheroids in Agitation-Based Cultures: A Scaffold-Free Cell Model That Sustains Long-Term Survival of Endothelial Cells
Source: Front Bioeng Biotechnol. 2021 Jun 9;9:649949. doi: 10.3389/fbioe.2021.649949 (PMC8219978; doi:10.3389/fbioe.2021.649949)
Supplement: Supplementary file 1 [file Data_Sheet_1.pdf]

Supplementary Material

1 Supplementary Methods

1.1 Flow cytometry

Heterotypic triple spheroids composed of HCC1954 tumor cells (TC), human dermal fibroblasts (hF), and HUVECs (EC) labeled with CellTracker Deep Red (Invitrogen), were collected at days 14 and 30 of culture and processed for assessment of the EC population by flow cytometry. Spheroids were washed in PBS, dissociated by incubation with collagenase (1.5 mg/ml, Collagenase type 2, Worthington Biochemical), for 20 min, followed by washing and two cycles of incubation with trypsin (0.25%, ThermoFisher), 10 min each. Spheroid dissociation was confirmed by visual inspection. Cells dissociated in the different steps were pooled together, washed in PBS and resuspended in 2% (v/v) fetal bovine serum (FBS) in PBS. The cell suspensions were filtered in a 30 µm cell strainer (CellTrics®, Sysmex) before analysis in BD FACSCelesta cytometer. EC were identified as CellTracker Deep Red positive cells. TC, fibroblasts, and EC, all unlabeled, were used as autofluorescence controls and EC labeled with CellTracker Deep Red (Invitrogen) as positive control.

2 Supplementary Figures Caption

Supplementary Figure 1. Experimental workflow for the establishment of long-term cultures of triple heterotypic spheroids. At day 0, human dermal fibroblasts as surrogate for cancer associated fibroblasts (hF) and HUVECs as surrogate for tumor endothelial cells (EC) were labeled with the fluorescent dyes PKH26 red and CellTracker Deep Red, respectively. Single cell suspensions of tumor cells (TC), hF and EC were mixed at the ratios specified and seeded into ultra-low adherence (ULA) 96-well plates. At day 2 of culture, triple heterotypic spheroids were transferred to shake flasks and cultured under orbital agitation (100 rpm). Agitation-based spheroid cultures were maintained up to 28 days (day 30 of culture).

Supplementary Figure 2. Composition, morphology, and cell viability of triple heterotypic spheroids generated from different breast cancer cell lines. **A), B)** & **C)** Phase contrast and fluorescence microscopy at day 2 of culture (in 96 ultra-low adherence well-plates), before transfer to agitation-based cultures. Spheroids composed of: breast cancer cells (TC); human dermal fibroblasts, labeled with PKH26 (hF, red); HUVEC, labeled with CellTracker Deep Red (EC, cyan), in a TC:hF:EC ratio of 1:1:1 (6000 cells per well). In C, live cells were stained with FDA (green) and dead cells with PI (red). Scale bar, 250 µm. **D)** Spheroid area at days 2 and 30 of culture (TC:hF:EC ratio of 1:1:1; 6000 cells per well). Data are presented as mean ± SD from 3 independent experiments, except for MCF-7 (N=2); in each experiment, at least 20 spheroids were quantified per condition. For BT474 and HCC9154, statistical analysis was performed by an unpaired t-test two-tailed (comparison between days 2 and 30 of culture, for each cell line, ***$p<0.001$ and *$p<0.05$). **E)** Immunofluorescence microscopy of BT474 heterotypic spheroids, at day 30 of culture. Spheroids

generated by assembly of BT474 tumor cells, hF (PKH26, red) and EC (CellTracker Deep Red, cyan) were probed for the EC marker, CD31 (green); nuclei were stained with DAPI. Scale bar, 100 µm. Representative cryosection from the 5-10 spheroids assessed in each of the 3 independent experiments performed.

Supplementary Figure 3. Effects of total cell number and cell ratio on the composition of triple heterotypic spheroids. **A)** Fluorescence microscopy at day 30 of culture (after 2 days of static culture followed by 28 days of agitation-based culture). Spheroids generated with different total cell/well, at distinct cell ratios of HCC1954 tumor cells (TC), human dermal fibroblasts (hF, labeled with PKH26, red) and HUVECs (EC, labeled with CellTracker Deep Red, cyan). Live cells were stained with FDA, green. Images are representative of 5-10 spheroids assessed per condition, in each of the 3 independent experiments performed. Scale bar, 250 µm. **B)** Fold change in EC area (normalized for the total spheroid area measured in the equatorial axis) relative to the respective ratio using 1000 cell/well; data are presented as mean ± SD from 3-4 independent experiments, except for 3000 cell/well (N=2); in each experiment, at least 20 spheroids were quantified per condition. Statistical analysis was performed by a one-way ANOVA followed by post-hoc Tukey's multiple comparison test (excluding the 3000 cell/well conditions); no significant differences found.

Supplementary Figure 4. Triple heterotypic spheroid EC composition along culture. Flow cytometry detection of the EC population of triple heterotypic spheroids, at days 14 and 30 of culture (after 2 days of static culture followed by 12 and 28 days of agitation-based culture, respectively). EC were detected by CellTracker Deep Red fluorescence. Data is presented as EC % relative to total cells, mean ± SD % for day 14 (2 and 3 independent experiments for 1:1:1: and 1:3:10, respectively) and as mean for day 30 (1 independent experiment); % relative to total cells.

Supplementary Figure 5. Triple heterotypic spheroid area, cell viability and composition along culture. Total spheroid area at day 2 and 30 of culture (after 2 days of static culture followed by 28 days of agitation-based culture); spheroids of HCC1954 tumor cells (TC), human dermal fibroblasts (hF) and HUVECs (EC), generated with distinct TC:hF:EC, and monotypic spheroids of TC (mono TC). **A)** all wells seeded at 6000 cell/well. **B)** all wells seeded with the number of TC used in triple heterotypic spheroids of TC:hF:EC 1:3:10. (*p=0.024 between day 2 and 30 in each condition; unpaired t-test two-tailed). Data in A) and B) are presented as mean ± SD from 3 independent experiments. In each experiment, at least 20 spheroids were quantified per condition. Statistical analysis was performed by an unpaired t-test two-tailed (comparison between day 2 and 30 of culture for each condition, *p<0.05,). C) Spheroids of HCC1954 tumor cells (TC), human dermal fibroblasts (hF) and HUVECs (EC, labeled with CellTracker Deep Red, cyan), generated with TC:hF:EC of 1:1:1 and 1:3:10, and monotypic spheroids of TC (mono TC); all seeded at 6000 cell/well. **C)** Fluorescence microscopy at day 30 of culture (after 2 days of static culture followed by 28 days of agitation-based culture). Live cells were stained with FDA (green) and dead cells with PI (red). Images are representative of 5-10 spheroids assessed per condition, for each of the 3 independent experiments performed. Scale bar, 250 µm.

Supplementary Figure 6. Characterization of endothelial cells in triple heterotypic spheroids, cultured for 30 days under static conditions. Immunofluorescent detection of the endothelial cell marker CD31 (green) and nuclei (DAPI, green) in 10 µm cryosections of spheroids of HCC1954 tumor cells, human dermal fibroblasts (hF) and endothelial cells seeded at TC:hF:EC of 1:1:1 and

1:3:10 (TC:hF:EC). Scale bar, 100 µm. Images are representative of the 5-10 spheroids assessed per condition, for each of the 3 independent experiments performed.

Supplementary Figure 7. Fibroblasts are essential for endothelial cell maintenance in heterotypic spheroids. **A)** Phase contrast and fluorescence microscopy at days 2 and 30 of culture (2 days in 96 ultra-low adherence well-plates followed by 28 days in agitation-based culture). Spheroids composed of HCC1954 tumor cells cells (TC) and HUVECs, labeled with CellTracker Deep Red (EC, cyan); TC: EC of 1:1 and 1:10 (6000 cells per well). Live cells were stained with FDA (green). Scale bar, 250 µm. **B)** Phase contrast and fluorescence microscopy at day 30 (2 days in 96 ultra-low adherence well-plates followed by 28 days in agitation-based culture) of spheroids composed of TC and EC (labeled with CellTracker Deep Red, cyan), cultured in fibroblast conditioned medium since day 0. Scale bar, 250 µm. Images from A) and B) are representative of the 5-10 spheroids assessed per condition, in each of the 2-3 independent experiments performed.

Supplementary Figure 8. Quantification of interleukin 6 (IL-6) in spheroid culture supernatants. Spheroids of HCC1954 tumor cells (TC), human dermal fibroblasts (hF) and HUVECs (EC), generated with TC:hF:EC of 1:1:1, 1:1:3 and 1:3:10, monotypic spheroids of TC and double spheroids of TC:hF 1:1; all seeded at 6000 cell/well. IL-6 concentration in the spheroid culture supernatants from day 30, quantified by ELISA. White bar represents fresh culture medium. Data are presented as mean ± SD from 2 independent experiments, except IL-6 in TC:EC 1:1 (N=1). ND, not detected.

Supplementary Figure 1

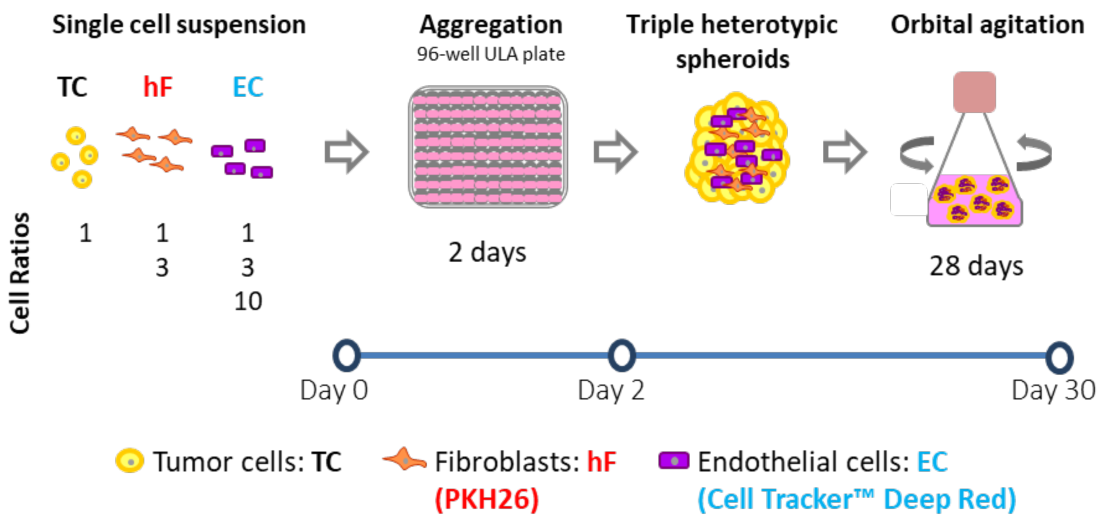

Supplementary Figure 2

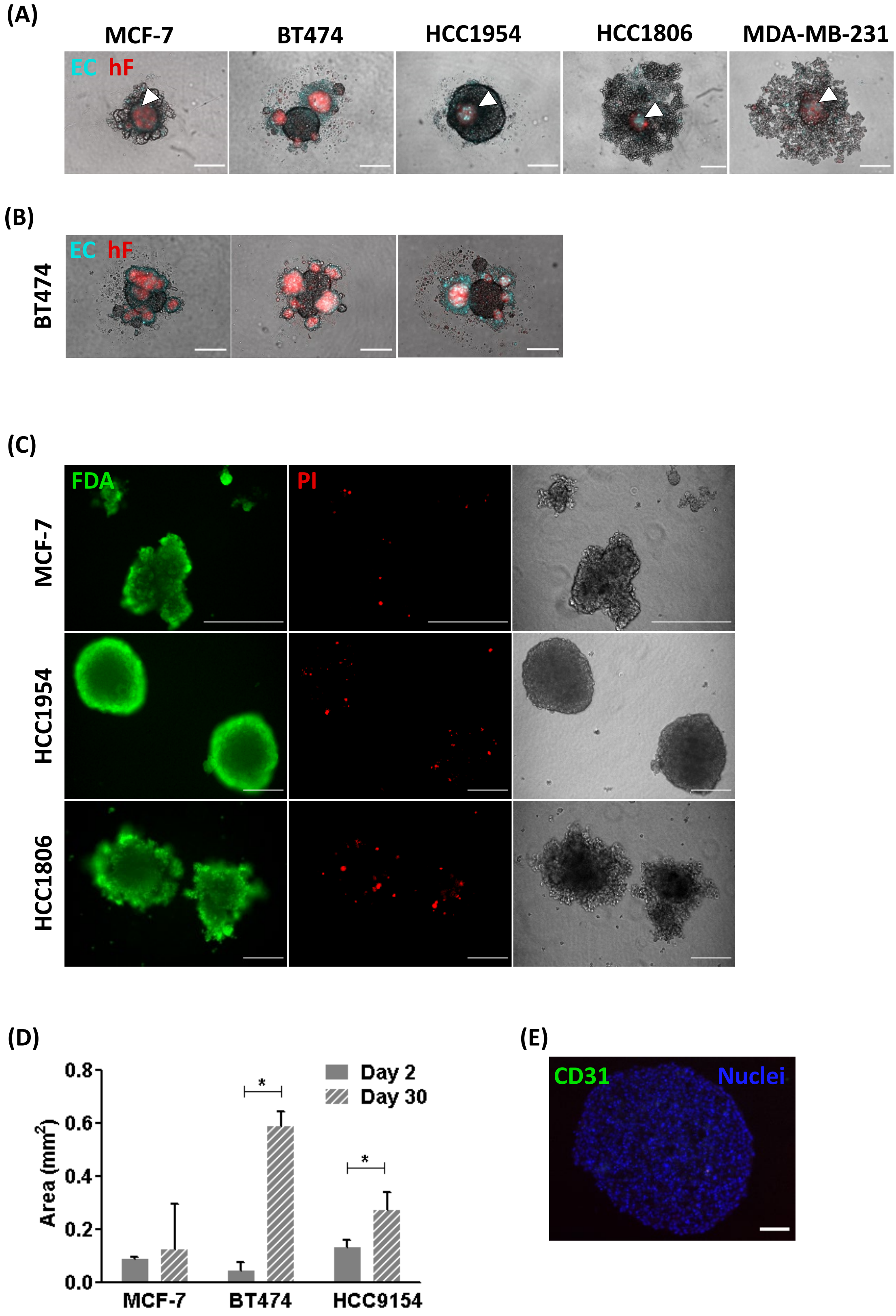

Supplementary Figure 3

(A)

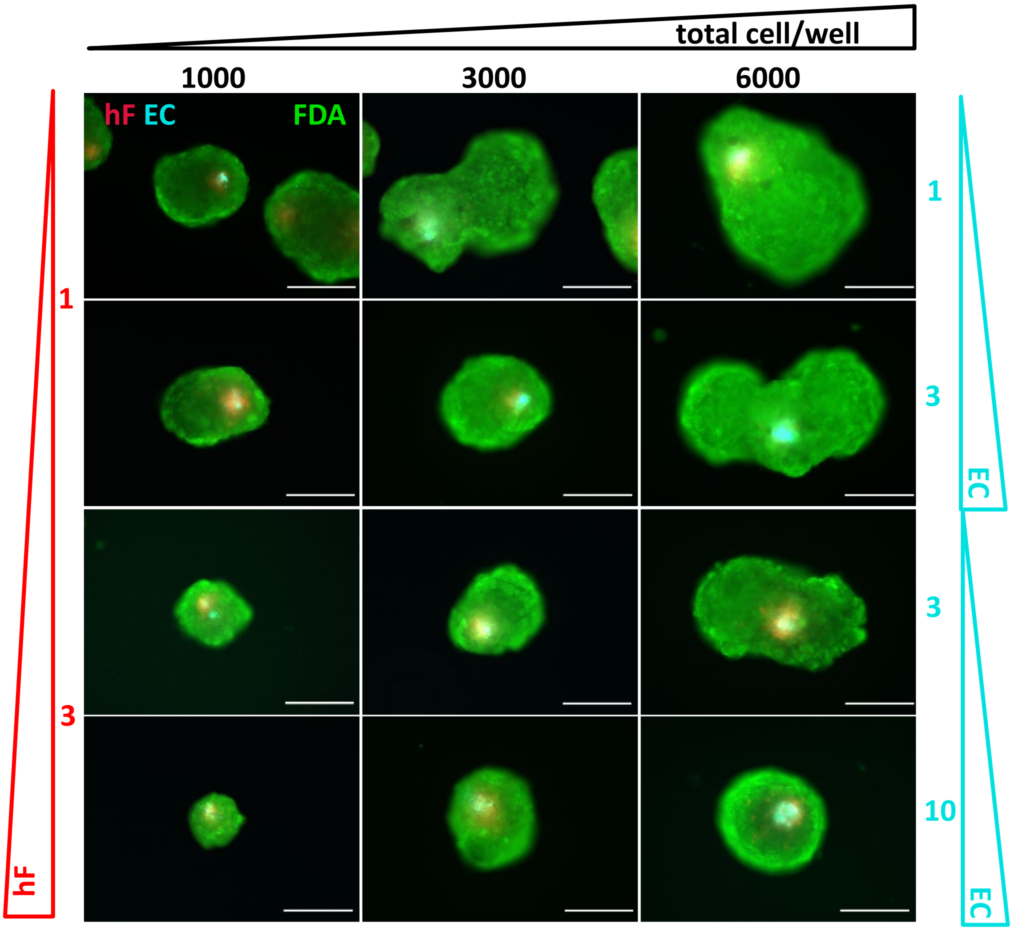

(B)

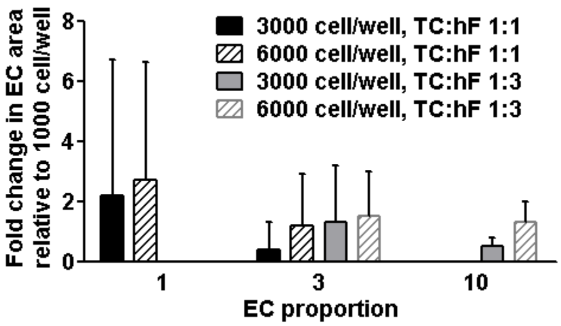

Supplementary Figure 4

	Day 14	Day 30
Cell Ratio	Mean ± S.D. (%)	Mean (%)
1:1:1	2.2 ± 0.3	2
1:3:10	3.9 ± 1.03	8

Supplementary Figure 5

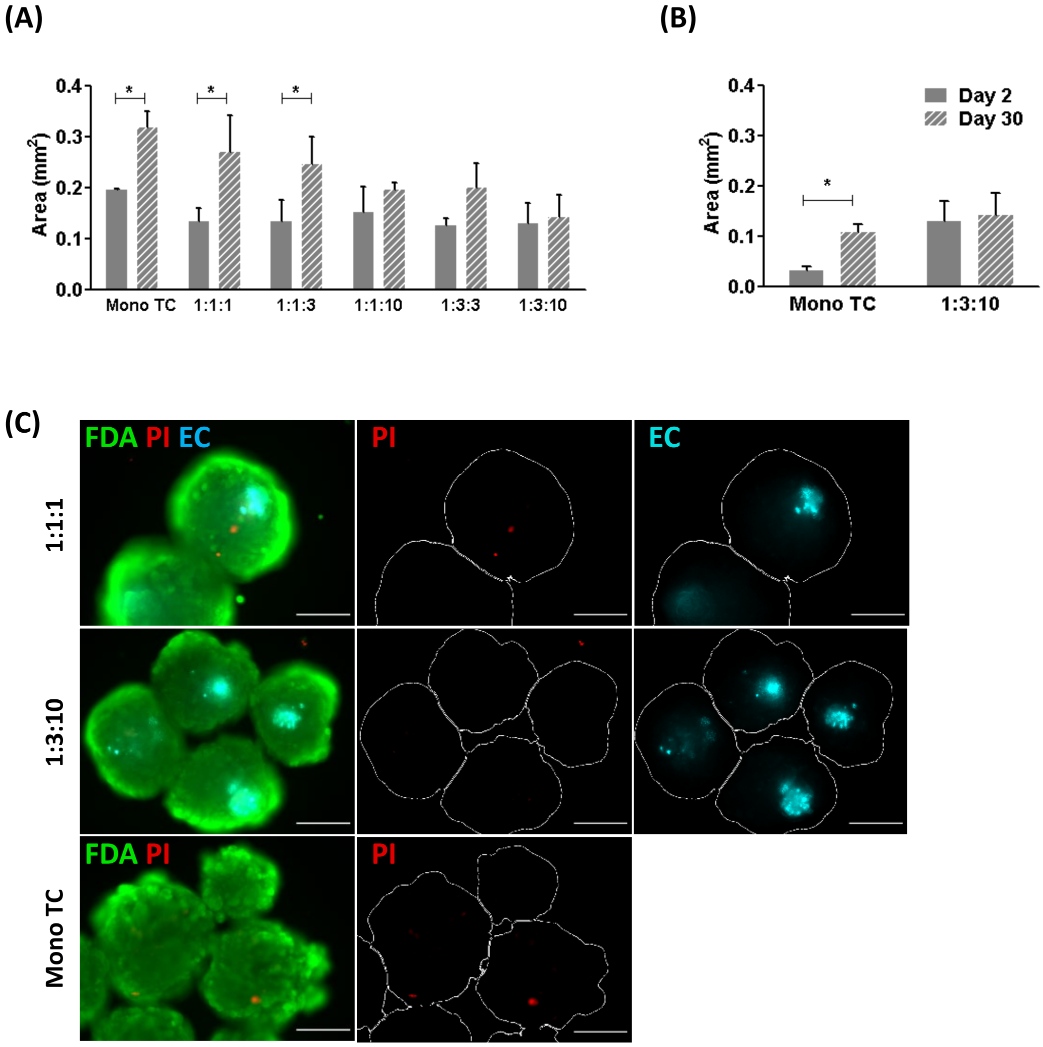

Supplementary Figure 6

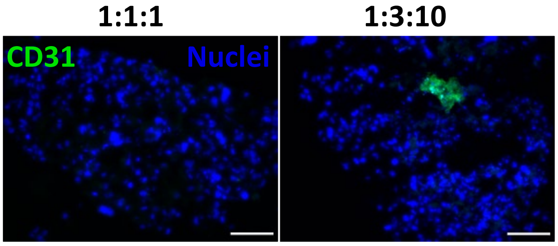

Supplementary Figure 7

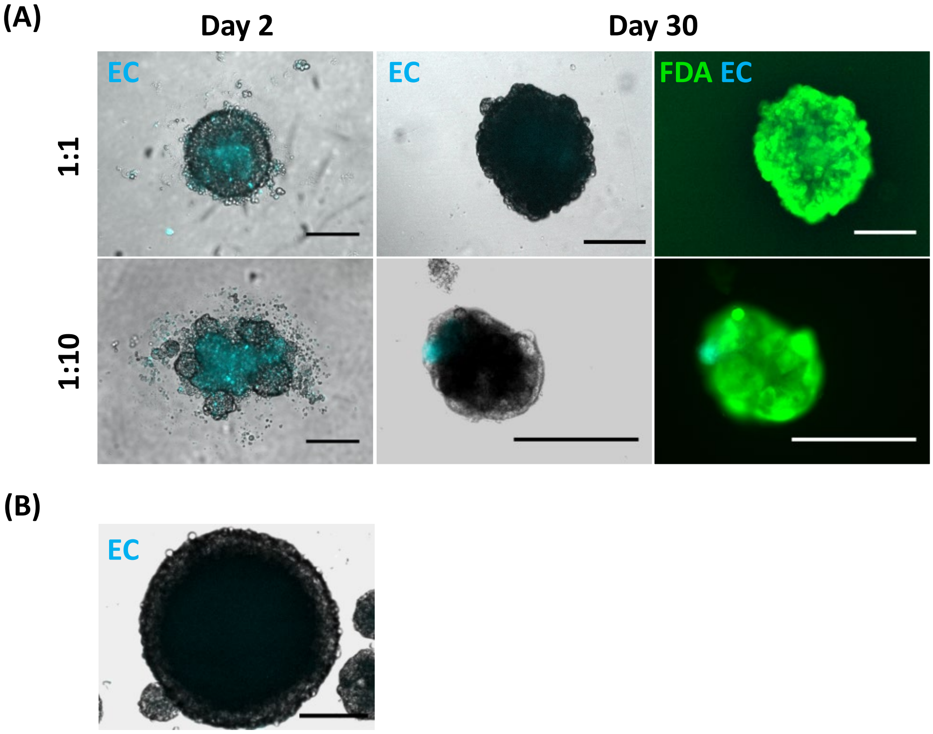

Supplementary Figure 8

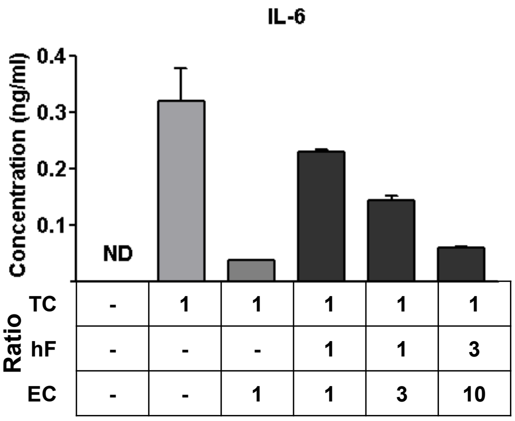

Ratio						
TC	-	1	1	1	1	1
hF	-	-	-	1	1	3
EC	-	-	1	1	3	10
